# Fatty Acids Rich Extract From *Clerodendrum volubile* Suppresses Cell Migration; Abates Oxidative Stress; and Regulates Cell Cycle Progression in Glioblastoma Multiforme (U87 MG) Cells

**DOI:** 10.3389/fphar.2018.00251

**Published:** 2018-03-20

**Authors:** Ochuko L. Erukainure, Nadia Ashraf, Asma S. Naqvi, Moses Z. Zaruwa, Aliyu Muhammad, Adenike D. Odusote, Gloria N. Elemo

**Affiliations:** ^1^Nutrition and Toxicology Division, Federal Institute of Industrial Research Oshodi, Lagos, Nigeria; ^2^Faculty of Pharmacy, Barrett Hodgson University, Karachi, Pakistan; ^3^Dr. Panjwani Center for Molecular Medicine and Drug Research, International Center for Chemical and Biological Sciences, University of Karachi, Karachi, Pakistan; ^4^Department of Biochemistry, Adamawa State University, Mubi, Nigeria; ^5^Department of Biochemistry, Ahmadu Bello University, Zaria, Nigeria; ^6^Analytical Division, Federal Institute of Industrial Research Oshodi, Lagos, Nigeria

**Keywords:** cancer, *Clerodendrum volubile*, oxidative stress, tumor migration, unsaturated fatty acids

## Abstract

Glioblastoma multiforme (GBM) is a malignant primary type of brain cancer with high proliferation and metastasis rates due to involvement of the microglial cell. It is resistant against available chemotherapy. Many strategic protocols have been developed but prognosis and patient life has not improved substantially. In this study, the anti-metastatic and antioxidant effect of fatty acids from *Clerodendrum volubile* leaves were investigated in U87-MG (Human Glioblastoma Multiforme) cell lines. The extracted fatty acids were incubated with U87-MG cells for 48 h. The anti-proliferative effect was determined by MTT assay, while apoptosis and cell cycle were analyzed with BD FACSCalibur. The transwell assay protocol was utilized in the analysis of cell migration and invasion. The treated cell lines were also assessed for reduced glutathione (GSH) level, catalase, superoxide dismutase (SOD) and lipid peroxidation. The fatty acid extract showed significant inhibitory activity on cell proliferation and cell cycle progression, mitigated oxidative stress, and suppressed migration and invasion in U-87 MG cell lines. These results give credence to the therapeutic potential of this plant against cancer, especially GBM.

## Introduction

Glioblastoma multiforme (GBM) is a malignant primary brain tumor common amongst the young kids with range of age < 13 years, characterized by high rates of proliferation, metastasis, and resistance to chemotherapy protocols (Brada et al., 2001; Puli et al., 2006; Markiewicz-Żukowska et al., 2013). These therapeutic failures represent a great difficulty in its treatment and management, leading to short survival rate, and loss of patient’s life quality (Puli et al., 2006). Its key epidemiologic risk includes age, sex, race, lifestyle behaviors, diet, and exposure to environmental factors including pollution from different sources (Efird, 2011).

Though epidemiology of glioblastoma has reported little or no occurrence in West Africa, changes in lifestyle particularly increased urbanization and adoption of western lifestyles and diet are however a major concern (Erukainure et al., 2016). An indigenous African diet often consists mainly of vegetable leaves, unrefined grains and spices, unlike the western counterpart consisting of sweetened desserts, saturated fats, and processed grains (Erukainure et al., 2016). Almost 30–40% of all cancers have been found to be preventive by healthy life style such as weight control, exercise, and physical activities (Potter and Potter, 1997; Jemal et al., 2011). Several studies have linked consumption of dietary fatty acids in diet with reduced brain cancer. Ketogenic diet characterized by high-saturated fats and low protein/carbohydrate, has proven to be effective during therapy and potent adjuvant management of malignancies in almost all types of cancers (Woolf and Scheck, 2015). Antal et al. (2014) reported increased sensitivity of glioblastoma cells to radiotherapy after treatment with arachidonic acid, docosahexaenoic acid, and γ-linolenic acid.

*Clerodendrum volubile* is among the common leafy vegetables in Southern part of Nigeria and it is well established for its medicinal use (Erukainure et al., 2010). It is indigenously known as *Obenetete* by the Itsekiris and Urhobos in the Delta of Niger. Commonly known as magic leaf, it is also used in the management and sometimes as adjunct for the treatment of diabetes mellitus, arthritis, rheumatism, ulcers, and many other diseases (Burkill, 1985). The phytochemicals of *C. volubile* and antioxidant activities have been reported (Adefegha and Oboh, 2010; Erukainure et al., 2011). Erukainure et al. (2014) isolated an iridoid glycoside from the leaves and reported its antioxidant activity in rats’ brain and hepatic tissues. In our previous study, we extracted dietary fatty acids from the leaves and investigated its effect on breast cancer cells (Erukainure et al., 2016). The fatty acids arrested cell cycle progression and down-regulated matrix metalloproteinase-9 in the breast cancer cells (Erukainure et al., 2016). Furthermore, molecular studies are required to prove the proclaimed medicinal uses of the extract of *C. volubile* leaves.

This present study aims to report the anti-proliferative, anti-oxidative, and anti-migratory and/or anti-metastatic activity of the fatty acid rich extracts from leaves of *C. volubile* on U87-MG cancer cells.

## Materials and Methods

### Plant Materials

Fresh *C. volubile* leaves, purchased from Ifon, Ondo State, Nigeria were identified and authenticated at the Department of Botany, University of Benin, Benin City, Nigeria (Voucher number: UBH_C284_).

The leaves were dried under shed, blended, and stored in deoxygenated container for further analysis (chemical, biochemical, and biological activities).

### Extraction of Fatty Acids

The blended leaves were subjected to methanol extraction, followed by fractionation with solvents of increasing polarity, as described by Erukainure et al. (2016).

The concentrated hexane fraction was subjected to methanolysis using the method described by Nickavar et al. (2003).

### Cell Cultures and Treatments

U87-MG cells were procured from American Type Culture Collection (ATCC). On arrival the ATCC instructions were followed and cells were submitted to the Bio-Bank of PCMD; ICCBS, University of Karachi, Karachi, Pakistan.

These cells were cultured in DMEM medium, 10% (v/v) fetal Bovine Serum (Sigma), L-glutamine 1% (v/v), penicillin 100 U/mL and streptomycin 100 μg/mL.

These newly seeded cells were kept in humidified incubator with 5% CO_2_.

### Cellular Cytotoxicity Analysis Using MTT as a Dye

The anti-proliferative activity of the extracted fatty acids against U87-MG cancer cells was evaluated in a 96-well plate using standard MTT [3-(4,5-dimethylthiazole-2-yl)-2,5-diphenyltetrazolium bromide] colorimetric assay, as described by Mosmann (1983).

Cells (1 × 10^4^ cells/mL) were seeded in 96-well plates.

After overnight incubation, the medium was replaced and 200 μL of fresh medium was added to each well, along with serial dilutions of the fatty acid extract (16, 32, 64, and 125 μg/mL, respectively).

Incubation at 48 h was done under same growth conditions, equal volume of the solution of dye MTT, 2 mg/mL, already prepared and preserved at -20°C was added to each well in triplicate manner.

After aspiration of nutrient media, 10% FBS and cell were then incubated for 4 h under same conditions as described for seeding the cells.

The nutrient media, 10% FBS 100 μL of DMSO were added to each well after aspiration of older media.

Absorbance was recorded at 570 nm wavelengths on a micro plate-reader (SoftMax PRO 4.3.1.LS, Molecular Devices, Sunnyvale, CA, United States).

The % inhibition was later calculated as follows:

(1) $$\%\;\text{Inhibition}\;\text{=}\;\text{100}\;\text{-}\;\left(\frac{\text{Mean}\;\text{absorbance}\;\text{of}\;\text{sample}}{\text{Mean}\;\text{absorbance}\;\;\text{of}\;\text{control}}\right)\;\times \;\text{100}$$

### Apoptotic Analysis by Propidium Iodide Flow Cytometry

Cells were seeded at 2 × 10^5^/mL/well in 24-well plate.

These cells were then incubated (under same growth conditions as described above) at 37°C overnight.

These cells were incubated with the extracted fatty acids at 37°C under 5% CO_2_ for 48 h. Cells treated with DMSO served as a negative control.

After incubations, cells were typsinized and centrifuged at 2500 rpm for 3–5 min. They were washed twice using phosphate buffer saline (PBS) before re-suspending in propidium iodide (PI) buffer.

One milliliter PI (0.5 mg/mL) was added for 1 min to the cells and their viability was analyzed with BD FACSCalibur.

### Fluorescent Activated Cells’ Cycle Analysis by Sorting (FACS)

U87-MG seeded in concentration of 1 million cells/mL/well using 6-well plate. The plate was incubated with the extracted fatty acid at 37°C for 48 h.

After incubation, the cells were then subjected to cell cycle analysis using flow cytometry on the BD Biosciences FACS machine, as described by Aliyu et al. (2013) and Erukainure et al. (2016).

The formula described by Pozarowski and Darzynkiewicz (2004) was used in calculating the duration of each phase.

### Migration and Invasion Assay

This was carried out according to the method described by Yao et al. (2012) with slight modifications, as U87-MG is a sensitive cell line and requires gentle handling.

5 × 10^4^ cells were seeded on top of the matrigel insert already prepared in top chamber with utmost care. Coating contained 150 μg matrigel on each membrane of top chamber.

The cells for both assays were trypsinized and resuspended in DMEM supplemented (700–900 μL) with 10% fetal bovine serum was gently pipetted down into the lower chambers. This was done in such a way that the surface of the media in lower chamber was just touching the lower side of the top matrigel-containing chamber.

The cells were incubated with the fatty acids extract of *C. volubile* in 120 μg/mL concentration at 37°C for 48 h for the migration and invasion assays, respectively. The cells at the top chambers were aspirated gently out.

Cells that adhered to the lower membrane of the inserts were fixed, and stained with solution of 20% methanol and 0.1% crystal violet.

They were subsequently counted and photographed with at 20–40× power-inverted microscope (Olympus Corp., Tokyo, Japan).

### Determination of Oxidative Stress Parameters

For this analysis the U87-MG cells were specifically counted in concentration of 1 million cells/mL, and added to 24-well plate and were then treated with the fatty acid extract of *C. volubile* at a concentration of 120 μg/mL.

These cells were analyzed for total protein (Lowry et al., 1951), reduced glutathione (GSH) level (Ellman, 1959), catalase activity (Chance and Maehly, 1955), superoxide dismutase (SOD) (Kakkar et al., 1984) activity, and also malondialdehyde (MDA) level, (Chowdhury and Soulsby, 2002).

### Inhibition of Chymotrypsin Activity

The α-chymotrypsin inhibitory activity of the extracted fatty acids was carried out as described by Cannell et al. (1988). Alpha-chymotrypsin (9 units/mL in 50 mM Tris-base buffer pH 7.6; Sigma Chemical Co., United States) was incubated with the fatty acid extract (2.5, 5.0, 10.0, and 20.0 μg/mL, respectively) for 20 min. A total of 100 pJ of substrate solution (*N*-Succinyl-phenyla1anine-pnitroanilide, 1 mg/mL of 50 mM Tris-Base buffer pH 7.6) was added to start the enzyme reaction. The absorbance of released p-nitroaniline was read at 410 nm.

### Statistics

To validate the significance of results, each experiment was repeated at least three times. Results were expressed as mean ± standard deviation (SD). One-way analysis of variance (ANOVA) was used in establishing statistical significance. While significant difference was established at *P* < 0.05 using LSD. Statistical analyses were carried out using SPSS for Windows, version 17.0 (SPSS Inc., Chicago, IL, United States).

## Results

**FIGURE 1:**
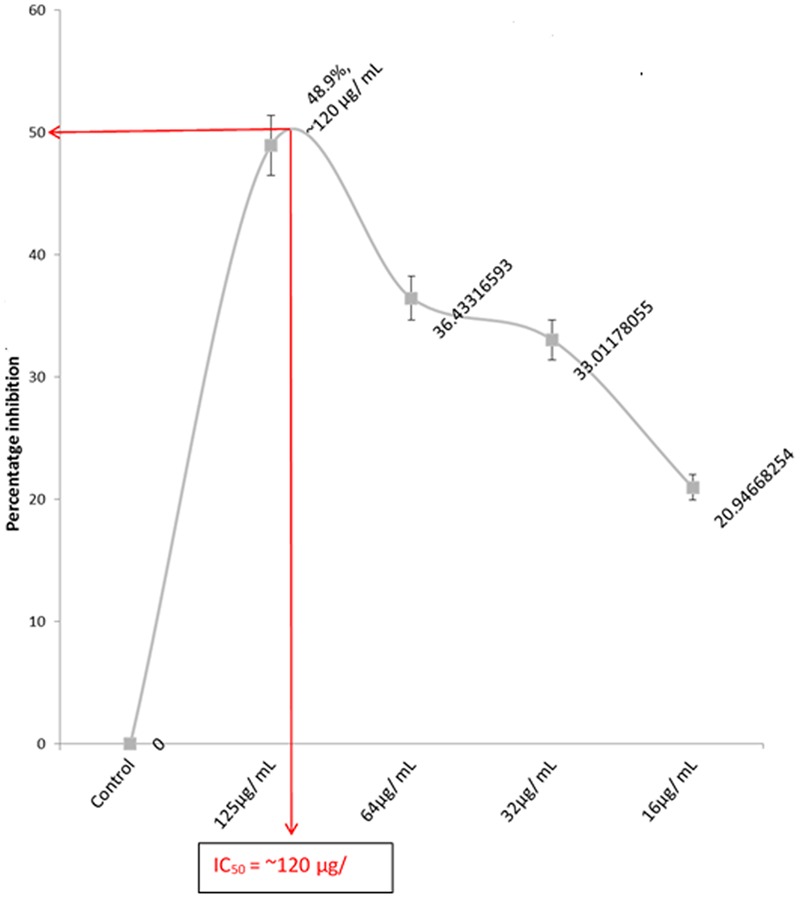
IC_50_ of the anti-proliferation effect of extracted fatty acids on U87 MG cell lines. Values = mean ± SD; *n* = 3.

A dose-dependent cytotoxic activity of the fatty acid extract was observed in U87-MG cell lines with an IC_50_ of ∼120 μg/mL as shown in **Figure 1**, indicating a potent anti-proliferative effect.

**FIGURE 2:**
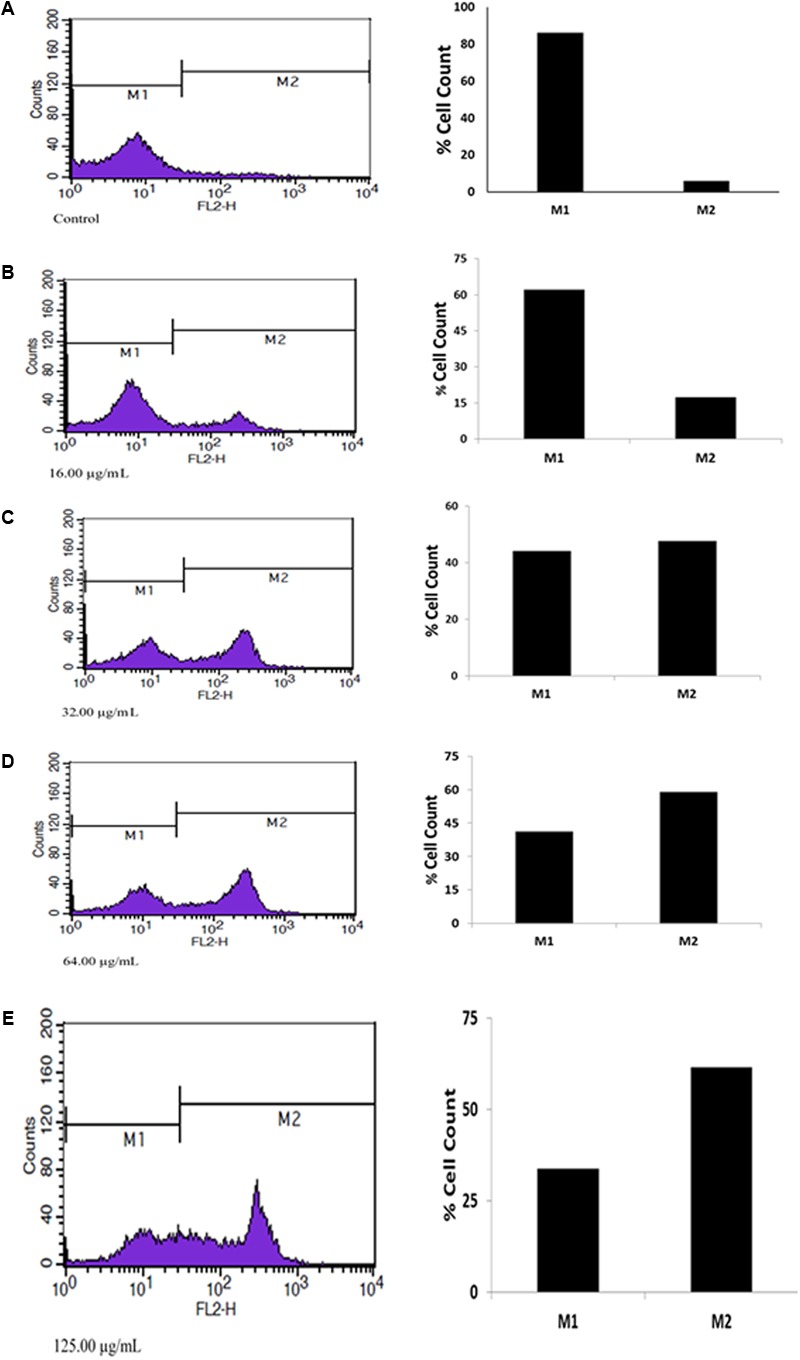
Apoptotic effect of extracted fatty acids on U87 MG cell lines. **(A)** Contol; **(B)** 16.00 μg/mL; **(C)** 32.00 μg/mL; **(D)** 64.00 μg/mL; **(E)** 125.00 μg/mL.

Apoptosis analysis by PI flow cytometry (**Figure 2**) revealed high cell deaths as the concentration increased, which is an indication of a dose-dependent anti-tumor activity of the fatty acid extract in U87-MG cancer cells.

**FIGURE 3:**
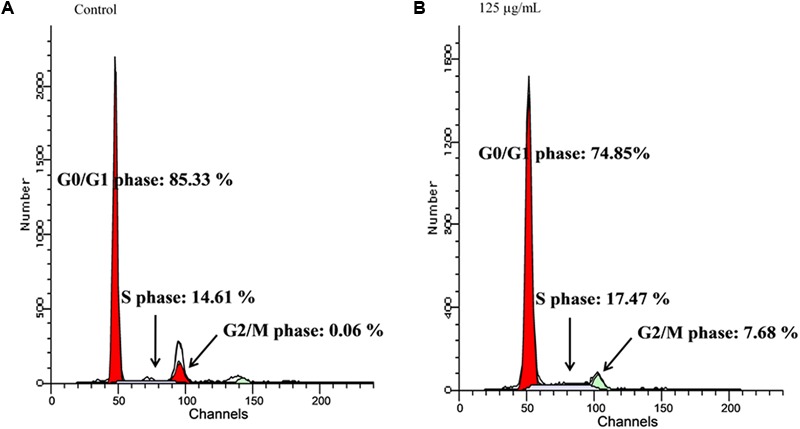
Effect of extracted fatty acids on cell cycle phase distribution of U-87 MG cell lines. **(A)** Control; **(B)** 125 μg/mL.

The quantity of DNA in each cancer cell population in G0/G1 and G2/M phases were significantly depleted in the fatty acids treated cells, as depicted in **Figure 3**. This was also observed with the simultaneous arrest of S phase of the cell cycle.

**FIGURE 4:**
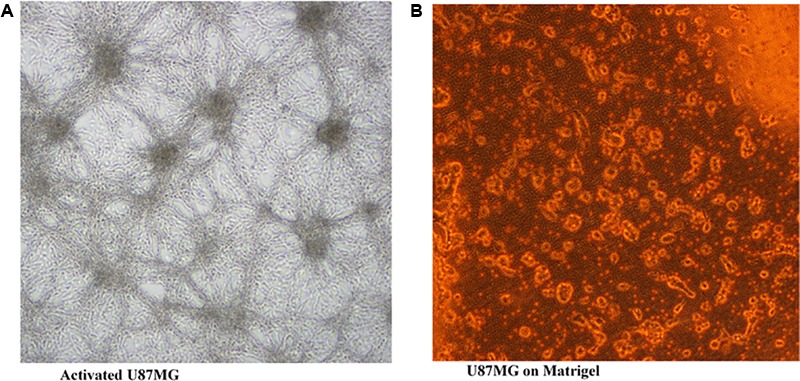
U87MG cells seeded on Matrigel (20× [2 mm]). **(A)** activated U87MG; **(B)** U87MG.

**FIGURE 5:**
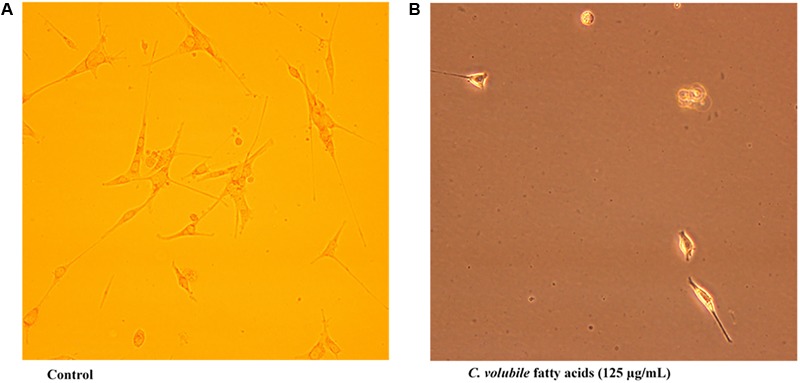
Effect of *C. volubile* fatty acids on cell migration and invasion of U87-MG (Human Neuronal Glioblastoma**)** cell lines (20× [2 mm]). **(A)** Control; **(B)** Fatty acids.

Transwell migration analysis revealed the migration of the untreated cells in all six parts of the chamber of 24-well transwell plate, as shown in **Figures 4, 5A**, indicating an occurrence of cell migration and/or invasion. However, the fatty acid treated cells showed little or no migration (**Figure 5B**), indicating a suppressive effect on cell migration and/or invasion.

**FIGURE 6:**
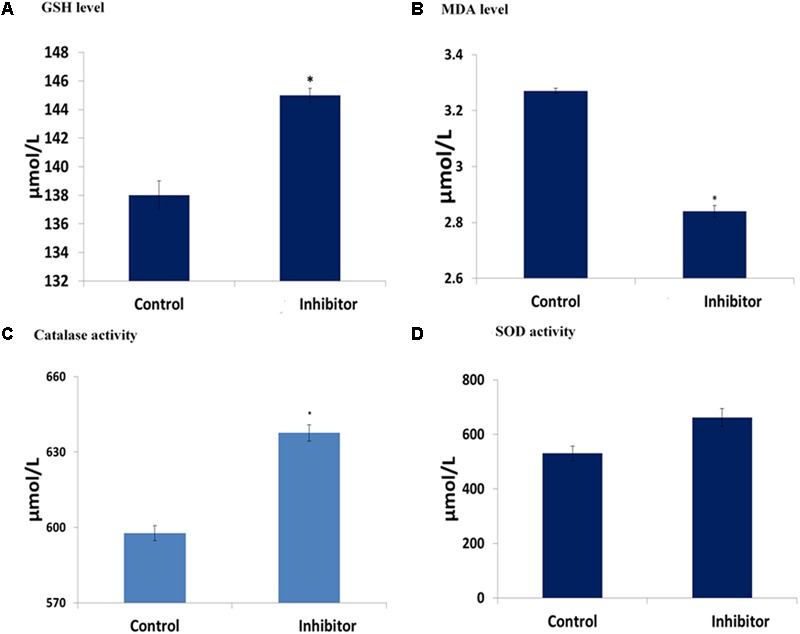
Antioxidative activities of *C. volubile* fatty acids in U87-MG (Human Neuronal Glioblastoma**)** cell lines. Values = mean ± SD; *n* = 3. **(A)** GSH level; **(B)** MDA level; **(C)** Catalase activity; **(D)** SOD activity.

Incubation of cells and fatty acid extract led to significant (*p* < 0.05) increase in GSH level with a concomitant decrease in MDA level, indicating an anti-oxidative activity as shown in **Figures 6A–D**. The significant (*p* < 0.05) increase as well as slight increase in catalase and SOD activities, respectively, further indicates the antioxidant potency of the fatty acids.

**FIGURE 7:**
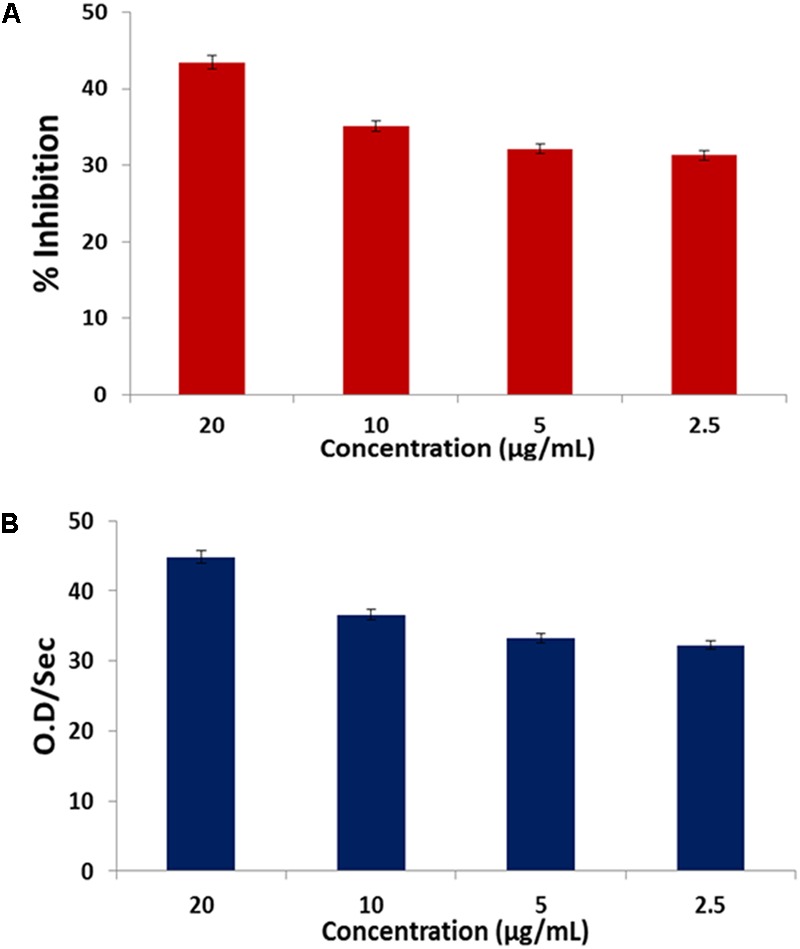
**(A)** Inhibitory effect of *C. volubile* fatty acids on α-chymotrypsin activity; **(B)** Effect of *C. volubile* fatty acids on kinetics (*V*_max_) of α-chymotrypsin. Values = mean ± SD; *n* = 3.

The fatty acids displayed a dose-dependent inhibitory effect on α-chymotrypsin activity and its kinetics as presented in **Figures 7A,B**.

## Discussion

Glioblastoma multiforme (GBM) has been recognized as a major brain tumor, and implicated in extensive invasion into neighboring brain tissue (Puli et al., 2006). This rapid invasion makes it difficult to treat especially while using surgical resection (Brada et al., 2001; Puli et al., 2006; Markiewicz-Żukowska et al., 2013). Hence, the need for an effective anti-proliferative agent that can suppress this invasion and/or migration with little or no side effects. In our previous study, we characterized the fatty acids extracted from *C. volubile* leaves using GC-MS to identify the fatty acid composition (Erukainure et al., 2016). Oleic acid was identified as the most abundant component, along with octadecanoic acid, n-hexadecanoic acid and 2-heptanone, 6-methyl in lesser quantities (Erukainure et al., 2016). In this present study, the suppressive effect of dietary fatty acids extracted from *C. volubile* was investigated on human glioblastoma multiforme cells with the aim of elucidating the possible molecular mechanism, along with its effect.

The cytotoxic effect of fatty acids on cancer cell lines has been reported in previous studies (Motaung et al., 1999). This corresponds with the observed cytotoxic effect of the extracted oil on U87-MG cells (**Figure 1**), thus indicating an anti-proliferative potential against GBM. This corresponds with our previous study of the anti-proliferative effect of *C. volubile* fatty against breast cancer, attributed to its high oleic acid concentration. The protective role of oleic acid against several cancers has been reported in previous epidemiological and animal studies (Carrillo et al., 2012). Thus, the observed anti-proliferative effect of the fatty acid extract, can be attributed to the high oleic acid content.

Novel natural therapies, targeted on induction of apoptosis in cancer cells, are gaining major interest. The role of apoptosis in cancer therapy is well emphasized in several studies. Most cancers manipulate and/or downregulates the antiapoptotic molecules, thereby evading this highly regulated program cell death (Koff et al., 2015). The observed apoptotic activity of the fatty acid extract as evident by the increased cell death (**Figure 2**), further indicates the anticancer potency of the fatty acids. This can be attributed to the arrest of S phase with concomitant depopulation of the DNA contents/cell population in G0/G1 as well as G2/M phases of the cell cycle (**Figure 3**). Cell cycle arrest has been implicated in the induction of apoptosis (Aliyu et al., 2013). Fauser et al. (2013) reported the induction of cell cycle by fatty acids.

Cell migration and invasion have been associated with the pathophysiology of cancer and in fact a major characteristic of malignant tumors and one of the key causes of cancer death (Bozzuto et al., 2010). In this study, the observed anti-migration, and invasion activity of the fatty acid extract on U87-MG cancer cells indicates a suppressive effect (**Figure 5**). This can be associated with the ability of the fatty acid enriched extract to inhibit α-chymotrypsin *in vitro* (**Figure 7**). Alpha-chymotrypsin has been implicated in the activation of metalloproteinases, and degradation of the extracellular matrix (ECM proteins), thus plays a major role in tumor growth and metastasis (Novak and Trnka, 2005; Mu et al., 2012). ECM acts as a physical barrier to migrating cells and must be degraded before the metastasis can occur. The *in vitro* inhibition of α-chymotrypsin (**Figure 7**) by the fatty acids therefore indicates a protective potential against ECM degradation. It can thus be suggested that the anti-metastatic action of the fatty acid extract could be facilitated by lowering the ability of glioblastoma cells to degrade the extracellular matrix components, by inhibiting the activities of α-chymotrypsin.

The implication of oxidative stress in the etiology of cancer has been reported (Erukainure et al., 2016). Oxidative stress arises due to an imbalance in the production of free radicals and the cell’s own antioxidant defenses (Erukainure et al., 2016). Often described as redox imbalance, it causes an impairment of normal cellular metabolism, which promotes malignancy, cancer initiation, and progression (Acharya et al., 2010). The observed reduced GSH level, SOD and catalase activities with concomitant increased MDA level in untreated U87-MG cells indicates an occurrence of oxidative stress (**Figures 6A–D**). Alteration of these enzymes has been reported in malignant cells and tumor tissues, thus suggesting an occurrence of redox imbalance in cancer cells (Barrera, 2012). The reversed levels and activities on following treatment with the fatty acids indicate an antioxidative potency against glioblastoma (**Figure 6**). Erukainure et al. (2016) reported a similar effect by same extract on MCF-7 cells, indicating the antioxidant protective effect of the leaf fatty acids against human invasive cancers. A remarkable association has been recognized between oxidative stress and increased uncontrollable drive of the G0/G1 and G2/M phases, leading to tumor metastasis and invasion (Pizarro et al., 2009; Chaudhary et al., 2013; Carrasco-Torres et al., 2017). This corresponds with the observed anti-oxidative activity, S phase arrest and concomitant depopulation of DNA contents/cell population in G0/G1 and G2/M phases of the cell cycle (**Figure 3**). This is also reflected by the decreased cell migration (**Figure 5**).

Fatty acids from *C. volubile* leaves could thus suppress tumor metastasis and/or invasion in human neuronal glioblastoma cells by the following mechanism: (1) attenuation of redox imbalance which inactivates the G0/G1 phase; (2) depopulation of DNA in the G0/G1 and G2/M phases with concurrent arrest of the S phase leading to inactivation of the cell cycle and apoptosis; and (3) Inhibition of α-chymotrypsin degradation of the extracellular matrix.

## Conclusion

The results of the current study suggest a suppressive effect of the fatty acid extract of *C. volubile* leaves against tumor metastasis and/or invasion in human neuronal glioblastoma cells, thus demonstrating their preliminary therapeutic potential. This fatty acid rich extract may thus be proposed as dietary supplements for the treatment, and prevention of such ailments.

## Author Contributions

OE, NA, AN, AM, GE, and MZ: conceived and designed the project. OE, AN, and NA: performed the MTT, apoptosis, transwell migration, and cell cycle assays. OE and AM: performed the oxidative stress assay. OE, AM, AO, and MZ: performed the statistical analysis. All authors were involved in the interpretation of results. OE, AM, GE, and MZ wrote the manuscript. All authors revised the manuscript.

## Conflict of Interest Statement

The authors declare that the research was conducted in the absence of any commercial or financial relationships that could be construed as a potential conflict of interest.
